# Ketogenic Diet Alleviates Colorectal Cancer by Attenuating Macrophage M2 Polarisation Triggered by Oncometabolite MMA Derived From the Gut Microbiota

**DOI:** 10.1111/cpr.70247

**Published:** 2026-06-17

**Authors:** Yang Lu, Bo Shi, Jinmiao Chen, Huihui Yao, Xiuwei Mi, Minke Shao, Yiyuan Zhao, Lijun Meng, Qingliang Tai, Junjie Chen, Xinyu Shi, Diyuan Zhou, Yizhou Yao, Songbing He

**Affiliations:** ^1^ Department of General Surgery The First Affiliated Hospital of Soochow University Suzhou Jiangsu China; ^2^ Advanced Molecular Pathology Institute of Soochow University and SANO, & SANO Medical Laboratories Suzhou Suzhou Jiangsu China; ^3^ Suzhou Sano Precision Medicine Ltd Suzhou Jiangsu China; ^4^ Department of General Surgery, Suzhou Ninth People's Hospital Suzhou Ninth Hospital Affiliated to Soochow University Suzhou Jiangsu China; ^5^ Department of Emergency Medicine The Fourth Affiliated Hospital of Soochow University Suzhou Jiangsu China; ^6^ Cancer Institute, Suzhou Medical College, Soochow University Suzhou Jiangsu China; ^7^ Suzhou Biomedical Industry Innovation Center & National Center of Technology Innovation for Biopharmaceuticals Suzhou Jiangsu China

## Abstract

Proposed mechanism of the ketogenic diet‐microbiota‐MMA‐immune axis in CRC. (Part 1) A ketogenic diet remodels gut microbiota homeostasis by depleting MMA‐producing bacteria, thereby reducing the accumulation of the oncometabolite (MMA). (Part 2) At the molecular level, MMA acts as a ligand that binds to Rap1, activating the downstream MAPK/ERK signalling cascade. This signalling event drives the transcriptional reprogramming of TAMs towards the pro‐tumorigenic M2 phenotype. (Part 3) Clinically, elevated serum MMA in CRC patients correlates with increased M2 macrophage infiltration in the tumour microenvironment and poor prognosis.

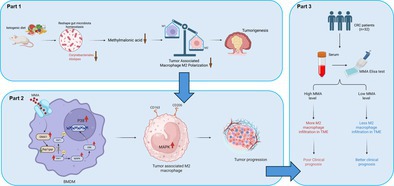

To the Editor,

1

Colorectal cancer (CRC) is one of the most ubiquitous cancers worldwide with approximately 1.93 million new cases each year [1]. Despite significant advances in the treatment of CRC in recent years, the prognosis for patients with advanced disease remains poor, highlighting an urgent need for novel preventive and therapeutic strategies. Emerging evidence has supported the promising potential of dietary interventions as safe and feasible adjuvant strategies to modulate tumour microenvironment (TME) [2], opening new avenues for optimising CRC management.

The ketogenic diet (KD), a high‐fat, low‐carbohydrate dietary therapy, has garnered increasing attention in recent years due to its potential therapeutic effects in tumours [3]. The KD could prevent the progression of CRC via inducing intratumor oxidative stress, and regulate the HDAC3/PKM2/NF‐κB65/p‐Stat3 axis [4]. Additionally, the KD modulates the function of immune cells within TME, thereby mediating significant anti‐tumour effects. Murphy et al. revealed that KD combined with immune checkpoint blockade (ICB) improved the function of tumour‐killing T cells' immunity to generate combinatorial anti‐tumour efficacy in a newly developed ICB‐refractory cancer model [5]. In our recent study, the KD inhibited pro‐tumour N2‐type tumour‐associated neutrophils (TANs) whilst promoting their polarisation towards the antitumor N1 type, which suppressed tumour growth and metastasis [6]. These studies illustrate the significant research value of the KD in improving CRC; however, our understanding of how the KD modulates other immune components in the TME and the precise regulatory mechanisms remain limited.

The gut microbiota promotes CRC progression and immune suppression through pro‐carcinogenic metabolites [7]. The gut microbiome is also crucial for the anti‐cancer effects of the ketogenic diet. Using a humanised mouse model, Tsenkova et al. confirmed that gut microbiota alterations drive the KD's anti‐cancer efficacy [8]. Notably, KD intervention increased faecal free stearic acid, which suppresses cancer directly and indirectly by inhibiting pathogenic colonic Th17 cells [8]. Furthermore, faecal microbiota transplantation (FMT) shows the KD exerts anti‐inflammatory and barrier‐protective functions by enriching beneficial bacteria (e.g., *Akkermansia muciniphila* ) [9]. This KD‐microbiota axis emphasises that diet‐responsive, tumour‐promoting bacteria (e.g., *Alistipes*, *Enterocloster*) and their metabolites are key mediators in CRC pathogenesis [10].

Tumour‐associated macrophages (TAMs) are one of the most prominent immune cells in the TME. The phenotype of macrophages undergoes dynamic changes and responds to various pro‐tumorigenic activities. In contrast to M1‐type, M2‐type TAMs can enhance the invasive capacity of tumour cells, suppress anti‐tumour immunity and promote tumour progression [11]. M2‐type TAMs tend to accumulate in the most active regions of CRC tissue and are usually positively correlated with poor prognosis [12, 13]. However, it remains unknown whether alterations in the gut microbiota and metabolites mediated by the KD can regulate TAMs polarisation.

In this study, we established a colitis‐associated cancer mouse model using AOM and DSS to further investigate the therapeutic efficacy of KD on CRC development. Mice were divided into normal diet control group (A/D) and ketogenic diet intervention group (A/D + KD), with the dietary intervention spanning the acute inflammation to chronic tumorigenesis phases (Figure 1A). Mice in the KD group were fed a strictly formulated ketogenic diet (Jiangsu Xietong Pharmaceutical Bio‐engineering Co. Ltd., catalogue no. XTKD01). The diet provided a macronutrient composition of 89.91% calories from fat, 9.99% from protein, and 0.1% from carbohydrates. The control group received a matched control diet. Both diets were provided *ad libitum*. All experimental procedures were approved by the Animal Ethics Committee of the First Affiliated Hospital of Soochow University and conducted in accordance with the Guide for the Care and Use of Laboratory Animals (Ethics Number: 2023127). Macroscopic examination of the mouse colons revealed that the AOM/DSS‐induced colorectal tumorigenesis was remarkably attenuated by KD intervention (Figure S1A). Quantitative analysis further corroborated this macroscopic observation as KD significantly reduced both the average number of tumours per mouse (*p* < 0.05, Figure S1B) and the mean tumour size (*p* < 0.01, Figure S1C) compared to the A/D group, which demonstrates that KD robustly suppresses colorectal tumour development in vivo.

**FIGURE 1:**
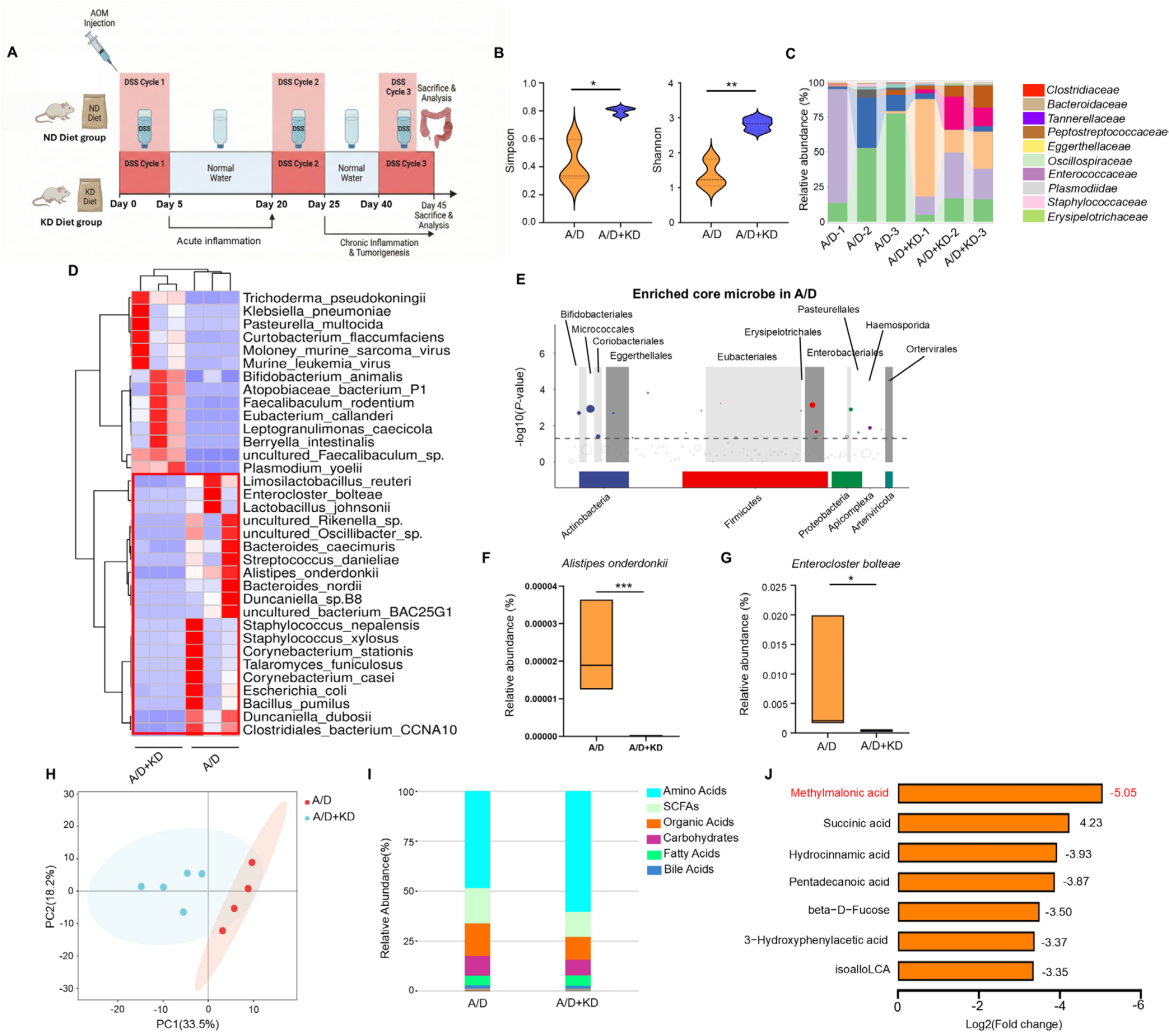
Ketogenic diet decreased the abundance of tumour‐promoting bacteria, resulting in reduced levels of the microbiota‐derived oncometabolite MMA. (A) Schematic representation of the AOM/DSS‐induced colitis‐associated colorectal cancer mouse model and the dietary intervention timeline. Mice were fed either a normal diet (A/D) or a ketogenic diet (A/D + KD). (B) Alpha diversity analysis (Simpson and Shannon indices) between the two groups. (C) Taxonomic composition at the family level demonstrating shifts in microbial abundance between the two groups. *n* = 3 for each group. (D) Heatmap clustering of species‐level abundance. (E) Manhattan plot identifying differentially abundant core taxa in A/D. (F–G) Quantitative analysis of specific tumour‐promoting bacteria. (H) Principal Component Analysis (PCA) of the faecal metabolome revealing clear separation between the A/D and A/D + KD groups. (I) Classification of identified metabolites in major compound classes. (J) Bar plot illustrating differentially abundant metabolites; methylmalonic acid (MMA) is identified as a significantly downregulated metabolite in the KD group (VIP > 1, *p* < 0.05). *, *p* < 0.05; **, *p* < 0.01, ***, *p* < 0.001.

To explore the effect of KD on gut microbiota change, we performed metagenomic sequencing on faecal samples of A/D and A/D + KD group. Alpha diversity analysis demonstrated that KD treatment significantly reversed the AOM/DSS‐induced reduction in microbial richness and evenness (*p* < 0.05 and *p* < 0.01, respectively: Figure 1B). Meanwhile, taxonomic profiling at the family level highlighted distinct compositional patterns, with visible shifts in the relative abundance of *Clostridiaceae*, *Bacteroidaceae* and *Tannerellaceae* (Figure 1C). Principal Coordinate Analysis (PCoA) of beta diversity analysis also revealed a distinct separation in the microbiota composition between the A/D and A/D + KD groups (Figure S2A). These findings indicate that KD induces a fundamental shift in the overall microbial community structure.

To pinpoint specific taxa driving these differences, we performed differential abundance analysis. Heatmap analysis displayed a distinct clustering pattern where the tumour‐promoting gut bacteria were highly abundant in the A/D model but suppressed by KD, which were highlighted in the red box (Figure 1D). Unexpectedly, the well‐known probiotic *L. reuteri* was found to be significantly enriched in the AOM/DSS mice, but its relative abundance was profoundly suppressed following the KD intervention. Furthermore, manhattan plots identified that the A/D + KD group showed a significant enrichment of beneficial core microbes, including members of *Bacteroidales*, *Lactobacillales* and *Bacillales*; conversely, the microbiome in the A/D group was enriched with microbes such as *Micrococcales*, *Coriobacteriales* and *Erysipelotrichales* (Figures 1E and S2B). Notably, we identified specific bacteria that have been reported to have a positive correlation with tumour progression. For example, the relative abundance of *Alistipes onderdonkii* and *Enterocloster bolteae*—species identified as correlating with the tumour‐bearing phenotype [14, 15]—was significantly elevated in the A/D group but markedly depleted in the A/D + KD group (Figure 1F,G). Collectively, these data indicate that the ketogenic diet inhibits CRC development, likely by reversing AOM/DSS‐induced dysbiosis and selectively reducing the abundance of tumour‐promoting bacteria.

Given the profound alterations in the gut microbiota composition, we further hypothesised that the therapeutic effects of KD might be mediated through shifts in the metabolites derived from gut microbiota. To test this, we performed untargeted metabolomic profiling on faecal samples from the A/D and A/D + KD groups. Principal Component Analysis (PCA) revealed a clear and robust segregation between the two groups along the first component (PC1, 33.5%), indicating that KD induces a fundamental reprogramming of the gut metabolome (Figure 1H). Meanwhile, to filter out noise unassociated with the grouping information, an Orthogonal Partial Least Squares Discriminant Analysis (OPLS‐DA) was employed and the OPLS‐DA plot demonstrated a distinct and clear separation between the A/D and A/D + KD groups, indicating significant metabolic alterations induced by KD (Figure S3A). Evaluation of metabolite classification further showed distinct variations in the relative abundance of major compound classes, including amino acids, short‐chain fatty acids (SCFAs) and organic acids, confirming a global metabolic shift (Figure 1I). To identify specific metabolites driving the anti‐tumour phenotype, a strict screening criterion was applied: a Variable Importance in Projection (VIP) score > 1.0 from the OPLS‐DA model and a *p*‐value < 0.05. After the first round of screening, we ranked the qualifying candidates according to their Fold Change (FC) and methylmalonic acid (MMA) emerged as the most prominently altered metabolite (|log2FC| = 5.05). (Figure 1J). Whilst other metabolites including succinic acid and hydro cinnamic acid also exhibited diet‐associated alterations, the depletion of MMA was the most striking feature linked to the KD. To integrate our microbiological and metabolomic findings, the correlation analysis was performed to examine the relationship between the specific bacterial taxa identified and the abundance of MMA (Figure S3B). The analysis revealed strong positive correlations between MMA concentration and the specific bacterial clusters that were enriched in the A/D group (e.g., *Alistipes*, *Bacteroides* and *Enterocloster* species), which suggested that the ketogenic diet may exert its protective effect by remodelling the tumour‐promoting microbiome to suppress the production of the oncometabolite MMA. Collectively, these findings identify MMA as a key microbiota‐derived metabolite that is markedly attenuated by KD intervention.

MMA was reported to have immunomodulatory actions in the TME [16]. After identifying it as a key oncometabolite markedly downregulated by the ketogenic diet, we aimed to confirm MMA's carcinogenic potential and clarify the underlying cellular mechanisms. By implanting MC38 tumour cells into C57BL/6 mice and then administering either MMA or PBS intraperitoneally, we created a subcutaneous syngeneic tumour model (Figure 2A). Notably, mice exposed to systemic MMA showed markedly higher ultimate tumour burden than the PBS‐treated controls (*p* < 0.01, Figure 2B,C). Furthermore, the expression of Ki‐67 in subcutaneous tumours was significantly increased after MMA intervention (Figure 2D). These phenotype data demonstrated that MMA accumulation promoted tumour progression in vivo.

**FIGURE 2:**
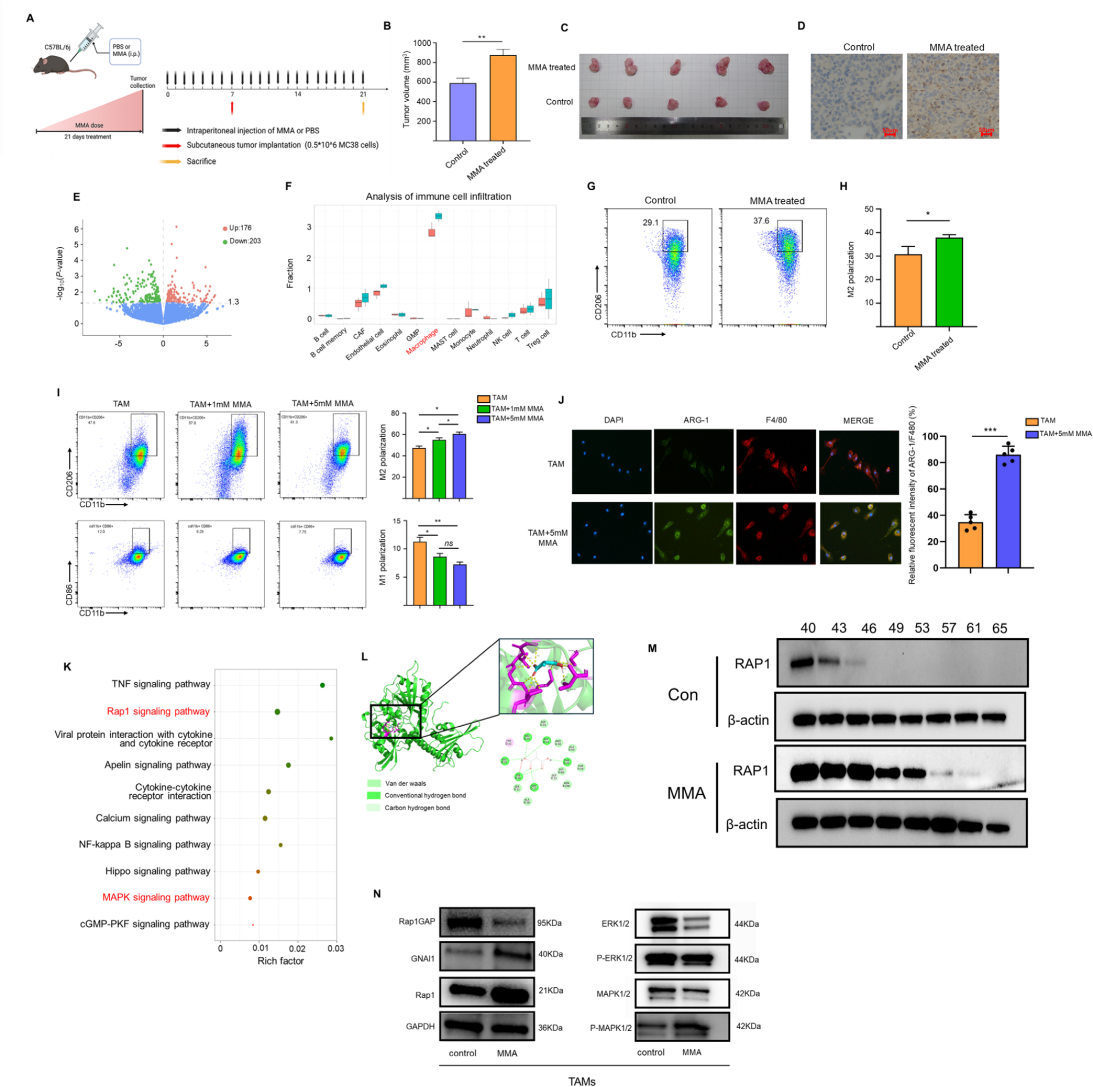
MMA promotes the phenotype and function of M2 macrophage polarisation in TME via Rap1 signalling pathway and MAPK signalling pathway. (A) Schematic of the in vivo experimental design using MC38 subcutaneous tumour implantation followed by intraperitoneal MMA injection. (B) Tumour volume anaysis showing accelerated progression in MMA‐treated mice. (C) Representative images of excised tumours. *n* = 5 for each group. (D) Immunohistochemical staining of KI‐67 on tumour sections. (E) Volcano plot of RNA‐seq data from control and MMA tumour tissues. (F) Immune cell infiltration analysis identifying macrophages as the predominantly enriched population in MMA‐treated tumours. (G–H) Flow cytometry analysis of tumour‐infiltrating CD206^+^ M2 macrophages. (I) Representative flow cytometry plots showing a dose‐dependent increase in CD206^+^ (M2 marker) cells and a decrease in CD86^+^ (M1 marker) cells upon MMA treatment (1 and 5 mM). *n* = 3 for each group. (J) Immunofluorescence staining showing enhanced expression and colocalization of ARG‐1 (green) in F4/80+ macrophages (red) following MMA treatment. Representative images were selected from three independent experiments, reflecting the average expression patterns of the groups. For quantitative analysis, the relative fluorescence intensity of ARG‐1 in F4/80‐positive cells was measured using ImageJ software from at least five randomly selected high‐power fields per sample. Statistical differences between the TAM and TAM + 5 mM MMA groups were evaluated using an unpaired two‐tailed Student's *t*‐test. Data are presented as mean ± SEM. (K) KEGG pathway analysis identifying “Rap1 signalling pathway” and “MAPK signalling pathway” as significantly enriched. (L) Molecular docking simulation predicting the direct binding of MMA to the active pocket of the Rap1 protein via hydrogen bonds. (M) Cellular Thermal Shift Assay (CETSA) validation of the interaction between MMA and Rap1. TAMs treated with vehicle (Con) or MMA were heated across a temperature gradient (40°C–65°C). The remaining soluble Rap1 and GAPDH were analysed by Western blotting. (N) Western blot analysis demonstrating increased levels of Rap1, p‐ERK1/2 and p‐MAPK1/2 in MMA‐treated TAMs. *, *p* < 0.05; **, *p* < 0.01, ***, *p* < 0.001.

Transcriptome analysis of subcutaneous tumours revealed distinct gene expression profiles between control and MMA groups (|log_2_FC| ≥ 2, *p* < 0.05, Figure 2E). Immune infiltration analysis showed a markedly reshaped landscape, with macrophages exhibiting the most significant increase in abundance (Figure 2F). Flow cytometry further confirmed that MMA treatment not only recruited macrophages but skewed their differentiation; CD11b+ CD206+ cells reached 37.8% in the MMA group versus 29.1% in controls (*p* < 0.05, Figure 2G,H). These results show that MMA recruits macrophages and encourages their polarisation into an M2 phenotype, which drives the advancement of colorectal cancer by establishing a tumour‐promoting microenvironment.

Further, we performed in vitro validation to determine whether MMA directly affects tumour associated macrophages (TAMs) to generate the phenotypic shift. We isolated bone marrow cells from C57BL/6 mice and differentiated them into bone marrow‐derived macrophages (BMDMs) within M‐CSF. After that, co‐culture system of MC38 cells and BMDMs was established using cell culture inserts with a 0.4‐μm pore size, which allowed us to obtain TAMs. We treated TAMs with different levels of MMA to detect the occurrence of cell polarisation (Figure S4A). Meanwhile, we used flow cytometry to confirm the purity of the F4/80^+^CD11b^+^ macrophage population (Figure S4B). MMA treatment induced significant alterations in cell surface markers. The number of CD206^+^ macrophages, which is a common M2 marker, went elevated in MMA dose‐dependent way, from 47.8% in the control group to 61.3% in the 5 mM MMA‐treated group (Figure 2I). On the other hand, MMA intervention reduced the expression of the M1 marker CD86, which indicated that MMA restrained anti‐tumour polarisation and encouraged alternative activation (Figure 2I). The immunofluorescence labelling also showed similar results of key phenotypic markers in macrophages. Figure 2J showed confocal microscopy pictures and quantitative statistics revealing the signal for ARG‐1 (green) was much larger in MMA‐treated macrophages compared to controls. However, F4/80 expression (red) was the same in all groups. Merged images demonstrated pronounced colocalization of ARG‐1 within F4/80^+^ cells specifically in the presence of MMA. RT‐PCR results also revealed that MMA treatment induced a profound, dose‐dependent upregulation in the relative mRNA expression of potent immunosuppressive cytokines and M2 markers, including CD206, IL‐10 and Arg‐1 (Figure S5A). Furthermore, the specific cytokine secretion levels of these polarised cells, as measured by ELISA, corroborated the transcriptomic findings; MMA significantly increased the levels of typical M2 factors (e.g., IL‐10 and TGF‐β) in the culture supernatant (Figure S5B). These in vitro findings provide compelling evidence that MMA functions as a potent signalling metabolite capable of directly polarising TAMs towards an immunosuppressive, tumour‐promoting M2‐like phenotype—thereby offering explanation for the accelerated tumour progression observed in vivo.

To definitively determine whether macrophages are the indispensable mediators of MMA‐induced tumour progression, rather than a concomitant phenomenon, we performed in vivo macrophage depletion assay using clodronate liposomes (CLD). Mice bearing MC38 subcutaneous tumours were treated with MMA in the presence or absence of intravenous CLD administration (Figure S6A). Whilst MMA administration remarkably accelerated tumour growth and increased tumour burden in macrophage‐intact mice, the depletion of macrophages completely abolished this pro‐tumorigenic effect. Both the tumour growth kinetic curves and the final endpoint tumour weights revealed no significant differences between the Control group and the CLD+ MMA co‐treatment group (Figure S6B–D). Meanwhile, we verified the depletion efficacy using flow cytometry. Notably, whilst CLD treatment alone did not significantly alter the baseline macrophage infiltration, it effectively abrogated the MMA‐induced accumulation of intratumoral macrophages (F4/80^+^ CD11b^+^ cells), bringing the infiltration levels back to the control group (Figure S6E,F). Collectively, these robust in vivo data provide direct evidence that MMA promotes colorectal cancer progression specifically through a macrophage‐dependent manner. This specific rescue experiment fundamentally consolidates our hypothesis, confirming that M2 macrophage polarisation is not merely a byproduct of the altered microenvironment, but rather the key causal mediator driving MMA‐induced tumour progression.

After in vivo and in vitro exploration, we performed RNA‐seq on TAMs treated with or without MMA to elucidate the specific molecular processes underlying the M2 macrophage polarisation. Principal Component Analysis (PCA) showed that the transcriptome profiles of the control group and the MMA‐treated group were distinct (Figure S7A). The GO enrichment analysis of the DEGs revealed that signal transduction ranked first amongst the biological processes that exhibited significant alterations (Figure S7B), which clearly demonstrates that MMA achieves the polarisation of macrophages towards the M2 phenotype by regulating downstream signal pathways. As a result, we mainly focused on KEGG pathways associated with intercellular signal transduction to ascertain the specific signalling cascades implicated. This KEGG analysis identified Rap1 and MAPK as the significant signalling pathways activated by MMA treatment (Figure 2K). Furthermore, we conducted molecular docking simulations to investigate whether MMA initiates this signalling cascade through interactions with Rap1, and the calculated binding free energy of MMA to Rap1 was −6.8 kcal/mol. Structural modelling revealed that MMA may occupy the active site of Rap1 and potentially form conventional hydrogen bonds with key amino acid residues essential for Rap1 function (Figure 2L). To further confirm whether MMA directly binds to the Rap1 protein, we performed a Cellular Thermal Shift Assay (CETSA) on TAMs. Western blot analysis revealed marked thermal stabilisation of Rap1 in the presence of MMA. Whilst endogenous Rap1 in the control group was largely degraded, MMA treatment significantly extended the stability of the Rap1 protein, with detectable bands remaining prominent up to 53°C and tracing even higher temperatures, which may suggest a direct physical interaction between MMA and the Rap1 protein (Figure 2M). Importantly, CETSA strictly provided direct physical evidence of MMA‐Rap1 interaction. The robust protection of Rap1 against heat‐induced denaturation following MMA treatment unambiguously confirms that MMA physically binds to and stabilises Rap1, establishing the direct molecular target of MMA. Whilst this establishes a direct MMA‐Rap1 binding axis, the precise upstream mechanism governing extracellular MMA uptake remains to be elucidated. As a metabolite, MMA might rely on specific membrane transporters (e.g., SLC family members) for cellular entry, which warrants further investigation to fully map this metabolic‐immune cascade.

Western blotting confirmed this signalling circuit at the protein level; MMA‐treated macrophages showed higher phosphorylation of downstream ERK1/2 and MAPK than controls, indicating an active MAPK cascade (Figure 2N). MAPK acts downstream of RAP1 [17], and its activation significantly induces TAM polarisation towards the M2 phenotype [18]. These results show MMA facilitates the Rap1/MAPK signalling complex. Finally, using the MAPK inhibitor SCH772984 in a rescue experiment, we demonstrated the Rap1/MAPK axis is essential for MMA‐induced M2 polarisation. Flow cytometry indicated MMA increased CD206^+^ M2 macrophages from 24.2% to 39.5%, whilst SCH772984 co‐treatment significantly reversed this to 27.9% (*p* < 0.05, Figure S8A,B). Our findings indicate MMA polarises M2 macrophages via the MAPK/ERK cascade, warranting further molecular research for drug development.

To ascertain the therapeutic significance of MMA on CRC progression, we expanded our study to clinical samples from colorectal cancer (CRC) patients. We hypothesised that the MMA‐macrophage axis found in our mouse models would be recapitulated in human pathology and correlate with disease progression. MMA levels are recognised as a reliable indicator of vitamin B12 deficiency [19], and a serum MMA concentration exceeding 0.40 μmol/L is defined as hypermethylmalonemia [20]. In the present study, we collected serum samples from 32 clinically confirmed CRC patients and quantified serum MMA levels using ELISA. Based on the cutoff value of 0.4 μmol/L, these patients were stratified into two subgroups: the high MMA group (20 patients) and the low MMA group (12 patients). As summarised in Table S1, there were no significant differences between the two groups regarding baseline clinicopathological characteristics, including age, gender, tumour location, depth of invasion (T stage), lymph node metastasis (N stage), distant metastasis (M stage), body mass index (BMI) and treatment plan (all *p* > 0.05). This well‐matched cohort ensures that subsequent analyses of the tumour microenvironment are minimally confounded by these baseline variables. The high‐MMA group showed substantially higher monocyte counts compared to the low‐MMA cohort, combined with a disruption in lymphocyte subsets and an altered CD4/CD8 ratio (Figure S9A). Further, we established the association between MMA and TAMs polarisation in the TME by conducting immunofluorescence labelling on tumour tissue slices from these patients. We employed CD68 as a universal macrophage marker and CD163 as a specific marker for the M2 phenotype. Confocal microscopy photos indicated that a greater number of CD68^+^CD163^+^ double‐positive cells were observed in high MMA patients, suggesting that the TAMs were converting into the M2 phenotype (Figure S9B). Conversely, tumour tissues from low‐MMA patients exhibited a scarcity of macrophages and less CD163 expression. Meanwhile, quantitative examination of the immunofluorescence data indicated a statistically significant increase in the percentage of M2 macrophages (CD163^+^CD68^+^ cells) in the High‐MMA group (*p* < 0.01, Figure S9C). We utilised linear regression analysis to find out how strong this link between MMA and TAMs infiltration was (Figure S9D). We detected a substantial positive connection (*R* = 0.749) between the blood concentration of MMA and the number of M2 TAMs in the tumour tissue. These clinical data greatly confirm our murine and in vitro findings, indicating a solid link between MMA accumulation, M2 macrophage enrichment and poorer clinical outcomes in CRC patients. This underscores the MMA‐macrophage axis as a prospective therapeutic target and prognosis marker.

In conclusion, our research identified that the gut microbiota‐derived MMA, a pro‐carcinogenic metabolite, directly induces M2 polarisation of TAMs and thereby accelerates CRC progression. Our results provided evidence that MMA functions as a robust signalling molecule that directly interacts with Rap1, therefore activating the MAPK pathway to promote an immunosuppressive TME. These findings underscore the significance of dietary control in cancer treatment and identify the Rap1‐MAPK axis and systemic MMA levels as viable targets for reprogramming the immune milieu in colorectal cancer.

## Author Contributions

Y.L. and S.H. wrote and polished the manuscript. Y.L., B.S., J.C., H.Y., X.M., M.S. and Y.Z. conducted experiments. Y.Z., Q.T., J.C., X.S., L.M., D.Z. and Y.Y. performed statistical analysis. Y.L., Y.Z. and D.Z. collected clinical data and samples. S.H. reviewed the writing and supervised the project. All authors have read and approved the final version of the manuscript and agree to be accountable for all aspects of the work.

## Funding

This work was supported by the Provincial‐level talent programme for National center of technology innovation for bio pharmaceuticals (NCTIB2024JS0101), the Jiangsu Province Chinese Traditional Medicine Science and Technology Development Plan Project (MS2025123), the Suzhou Basic Research Pilot Project (SSD2024047), the Suzhou Medical College‐QiLu Medical Research Programme of Soochow University (24QL200103), the Boxi Clinical Research The First Affiliated Hospital of Soochow University (BXLC2024022), the Prof. Changgeng Ruan's Research and Innovation Fund for Graduate Students of the First Affiliated Hospital of Soochow University (RKYCX202405, RSJCX202510) and the Graduate Cross‐Innovation Programme Suzhou Medical College of Soochow University (20244232055).

## Conflicts of Interest

The authors declare no conflicts of interest.

## Supporting information


**Figure S1:** Ketogenic diet suppresses colon tumour growth in an AOM/DSS‐induced mouse model. (A) Representative macroscopic photographs of excised colons from A/D and A/D + KD mice. Yellow arrows indicate distinct tumour nodules. Scale bar, 1 cm. (B and C) Statistical quantification of tumour number per mouse and average tumour size (mm). Data are presented as mean ± SEM. *p < 0.05, ***p < 0.01.
**Figure S2:** Ketogenic diet profoundly reshapes the gut microbiome composition in the AOM/DSS model. (A) Principal coordinates analysis (PCoA) plot demonstrating distinct clustering and separation of gut microbiota profiles between the A/D and A/D + KD groups. (B) Manhattan plot illustrating the significantly enriched core microbial taxa in the A/D + KD group. The *x*‐axis displays the major phyla, and the *y*‐axis represents the significance level.
**Figure S3:** Ketogenic diet alters the faecal metabolomic profile and its correlation with gut microbiota. (A) Orthogonal projections to latent structures discriminant analysis (OPLS‐DA) score plot based on faecal metabolomics data, showing clear metabolic separation between the A/D and A/D + KD groups. (B) Mantel test analysis demonstrating the correlation between the key metabolite (Methylmalonic acid) and the significantly altered gut microbial species.
**Figure S4:** Construction and verification of the in vitro tumour‐associated macrophage (TAM). (A) Schematic illustration of the experimental workflow. Bone marrow cells were isolated from the tibia and femur of mice and differentiated into bone marrow‐derived macrophages (BMDMs) in the presence of M‐CSF for 7 days. BMDMs were then co‐cultured with MC38 colon cancer cells for 24 h to generate TAM‐like cells, followed by treatment with or without MMA for 48 h prior to marker analysis. (B) Flow cytometric analysis characterising the purity of BMDMs and TAMs. Cells were stained with macrophage surface markers CD11b and F4/80. *n* = 3 for each group.
**Figure S5:** MMA dose‐dependently promotes M2 macrophage polarisation in vitro. (A) Relative mRNA expression levels of typical M2 macrophage markers (CD206, IL‐10 and Arg‐1) in TAMs treated with 0, 1 mM or 5 mM MMA. (B) Concentrations of secreted cytokines (IL‐10 and CCL22) in the cell culture supernatants, as measured by ELISA. Data are presented as mean ± SEM. *p < 0.05, **p < 0.01.
**Figure S6:** Systemic macrophage depletion abrogates the MMA‐induced promotion of tumour growth in vivo. (A) Schematic illustration of the experimental design. C57BL/6J mice bearing MC38 subcutaneous tumours were randomised and treated with MMA (200 mg/kg, i.p., daily) or vehicle control, with or without systemic macrophage depletion using intravenous clodronate liposomes (CLD, 200 μL/mouse) at the indicated time points. (B) Tumour growth curves depicting the tumour volumes of the indicated groups measured over time. (C) Representative macroscopic images of the excised MC38 tumours from each group at the experimental endpoint. (D) Quantitative analysis of the final tumour weights at the endpoint. (E) Representative flow cytometry plots showing the infiltration of tumour‐associated macrophages (TAMs) in the tumour microenvironment. (F) Statistical quantification of the percentage of TAMs (defined as F4/80^+^ and CD11b^+^ cells) within the total live CD45^+^ leukocyte population. Data are presented as mean ± SEM (*n* = 5 mice per group). Statistical significance was determined by one‐way ANOVA with Tukey's post hoc test. *p < 0.05, **p < 0.01; ns, not significant.
**Figure S7:** RNA‐seq analysis reveals altered signalling pathways in MMA‐treated TAMs. (A) Principal component analysis (PCA) plot of RNA‐sequencing data showing the distinct separation between the control group (con) and the MMA‐treated group (MMA) (*n* = 3 for each group). (B) Gene Ontology (GO) enrichment analysis of the differentially expressed genes (DEGs). The bar chart displays the top enriched biological processes. The numbers adjacent to the bars indicate the count of enriched genes in each pathway. The “signal transduction” pathway (highlighted in red) was identified as a significantly regulated process.
**Figure S8:** SCH772984 reverses MMA‐induced M2 polarisation of TAMs. (A) Representative flow cytometry plots showing the expression of CD11b and CD206 in TAMs. Cells were treated with MMA alone or in combination with the inhibitor SCH772984. (B) Quantification of M2 polarisation (percentage of CD11b and CD206 positive cells) across the indicated groups. *n* = 3 for each group. Data are presented as mean with SEM. Statistical significance was analysed using one‐way ANOVA. *, *p* < 0.05; **, *p* < 0.01.
**Figure S9:** MMA is closely associated with poor clinical prognosis and tumour‐associated macrophage infiltration in CRC patients. (A) Analysis of clinical blood samples showing that patients with High serum MMA levels exhibit significantly higher monocyte counts and altered CD4/CD8 ratios compared to the Low MMA group. (B) Representative immunofluorescence images of human CRC tissue sections stained for CD68 (red, macrophage marker) and CD163 (green, M2 marker). (C) Quantitative analysis showing a significantly higher percentage of M2 macrophages in the High MMA group. (D) Linear regression analysis revealing a strong positive correlation (*R* = 0.749) between serum MMA concentration and the density of infiltrating M2 macrophages in tumour tissues. *, *p* < 0.05; **, *p* < 0.01, ***, *p* < 0.001.
**Table S1:** Comparison of clinical characteristics according to serum MMA Levels in CRC patients.

## Data Availability

The data that support the findings of this study are available from the corresponding author upon reasonable request.
