# Supplementary material for: Ketogenic Diet Alleviates Colorectal Cancer by Attenuating Macrophage M2 Polarisation Triggered by Oncometabolite MMA Derived From the Gut Microbiota
Source: Cell Prolif. 2026 Jun 17:e70247. Online ahead of print. doi: 10.1111/cpr.70247 (PMC13325913; doi:10.1111/cpr.70247)
Supplement: Supplementary file 1 — Figure S1: Ketogenic diet suppresses colon tumour growth in an AOM/DSS‐induced mouse model. (A) Representative macroscopic photographs of excised colons from A/D and A/D + KD mice. Yellow arrows indicate distinct tumour nodules. Scale bar, 1 cm. (B and C) Statistical quantification of tumour number per mouse and average tumour size (mm). Data are presented as mean ± SEM. *p < 0.05, ***p < 0.01. Figure S2: Ketogenic diet profoundly reshapes the gut microbiome composition in the AOM/DSS model. (A) Principal coordinates analysis (PCoA) plot demonstrating distinct clustering and separation of gut microbiota profiles between the A/D and A/D + KD groups. (B) Manhattan plot illustrating the significantly enriched core microbial taxa in the A/D + KD group. The x‐axis displays the major phyla, and the y‐axis represents the significance level. Figure S3: Ketogenic diet alters the faecal metabolomic profile and its correlation with gut microbiota. (A) Orthogonal projections to latent structures discriminant analysis (OPLS‐DA) score plot based on faecal metabolomics data, showing clear metabolic separation between the A/D and A/D + KD groups. (B) Mantel test analysis demonstrating the correlation between the key metabolite (Methylmalonic acid) and the significantly altered gut microbial species. Figure S4: Construction and verification of the in vitro tumour‐associated macrophage (TAM). (A) Schematic illustration of the experimental workflow. Bone marrow cells were isolated from the tibia and femur of mice and differentiated into bone marrow‐derived macrophages (BMDMs) in the presence of M‐CSF for 7 days. BMDMs were then co‐cultured with MC38 colon cancer cells for 24 h to generate TAM‐like cells, followed by treatment with or without MMA for 48 h prior to marker analysis. (B) Flow cytometric analysis characterising the purity of BMDMs and TAMs. Cells were stained with macrophage surface markers CD11b and F4/80. n = 3 for each group. Figure S5: MMA dose‐dependently promotes M2 [file CPR-9999-e70247-s001.docx]

**Supplementary Materials**


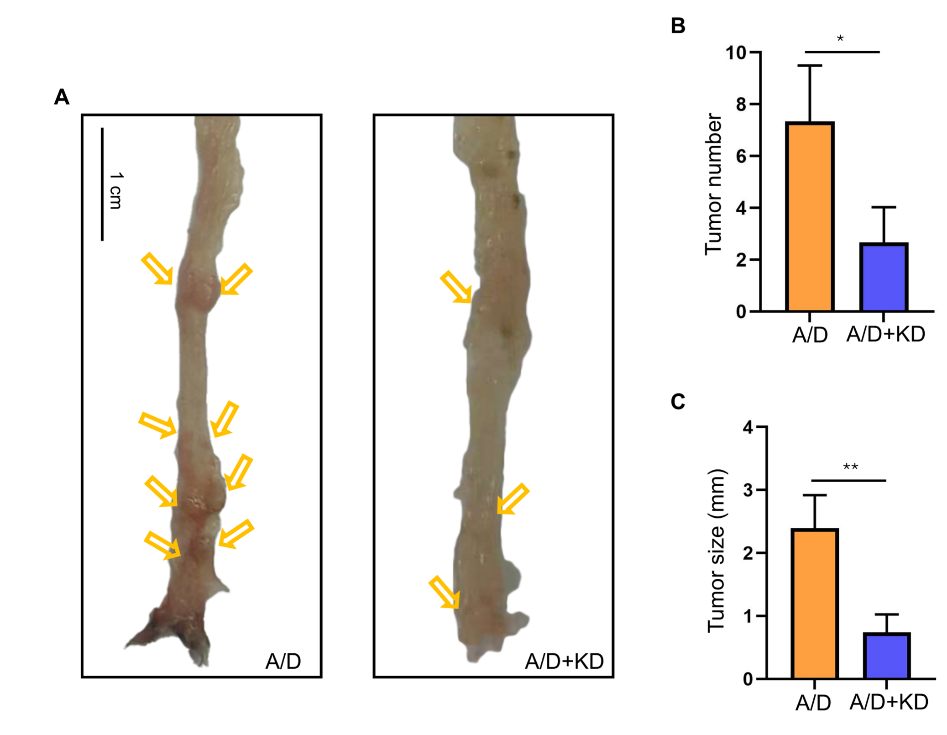


**Figure S1. Ketogenic diet suppresses colon tumor growth in an AOM/DSS-induced mouse model. (A)** Representative macroscopic photographs of excised colons from A/D and A/D+KD mice. Yellow arrows indicate distinct tumor nodules. Scale bar, 1 cm. **(B-C)** Statistical quantification of tumor number per mouse and average tumor size (mm). Data are presented as mean ± SEM. **P* < 0.05, ****P* < 0.01.

**
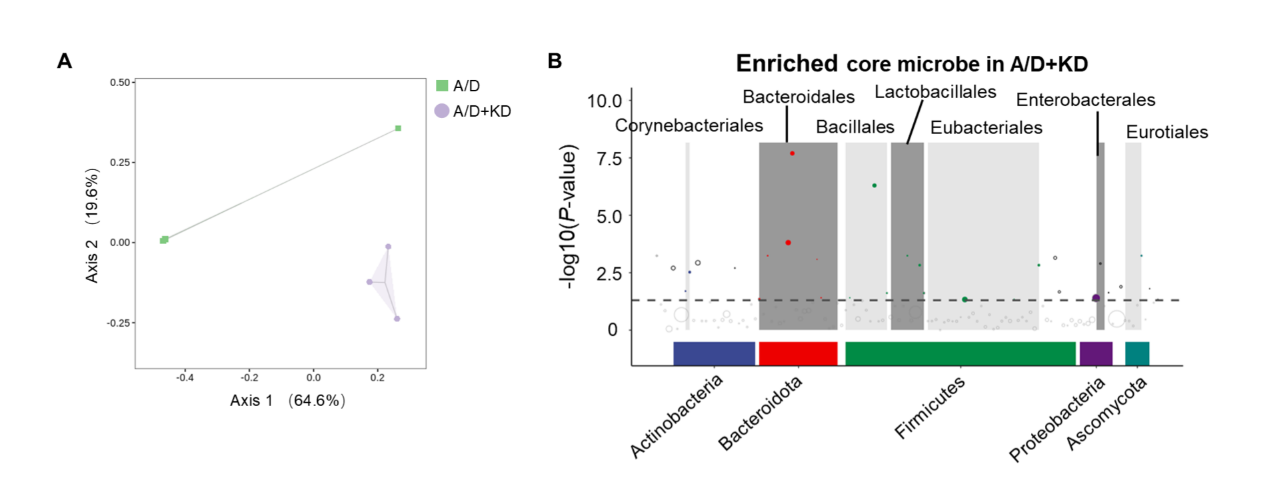
**

**Figure S2. Ketogenic diet profoundly reshapes the gut microbiome composition in the AOM/DSS model. (A)** Principal coordinates analysis (PCoA) plot demonstrating distinct clustering and separation of gut microbiota profiles between the A/D and A/D+KD groups. **(B)** Manhattan plot illustrating the significantly enriched core microbial taxa in the A/D+KD group. The x-axis displays the major phyla, and the y-axis represents the significance level**.**

**
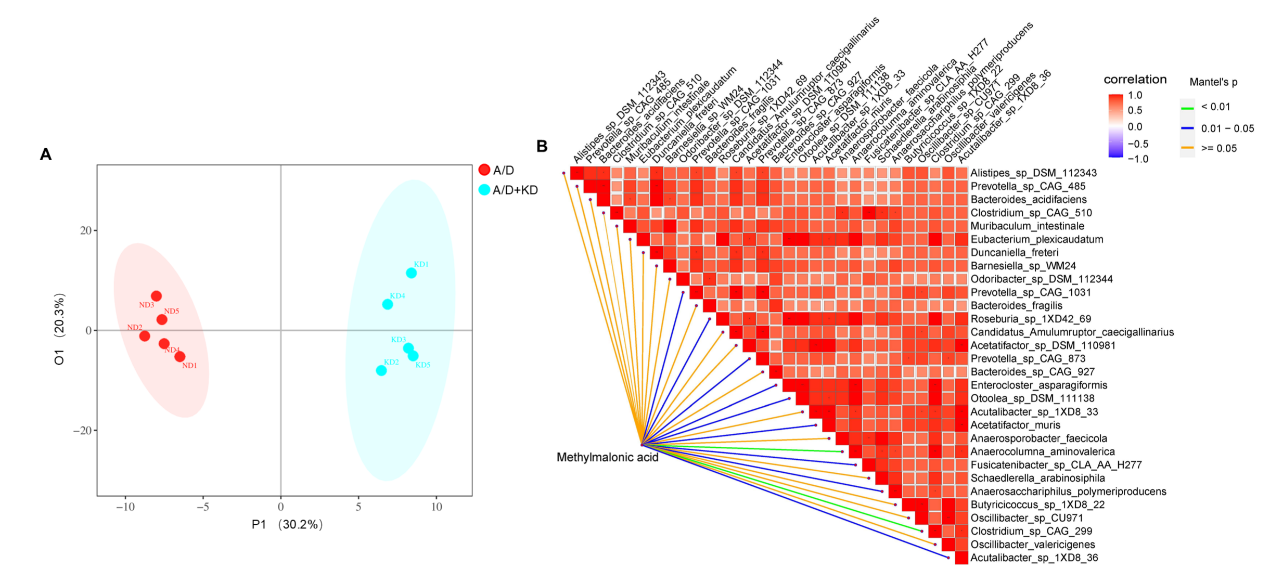
**

**Figure S3. Ketogenic diet alters the fecal metabolomic profile and its correlation with gut microbiota. (A)** Orthogonal projections to latent structures discriminant analysis (OPLS-DA) score plot based on fecal metabolomics data, showing clear metabolic separation between the A/D and A/D+KD groups. **(B)** Mantel test analysis demonstrating the correlation between the key metabolite (Methylmalonic acid) and the significantly altered gut microbial species.


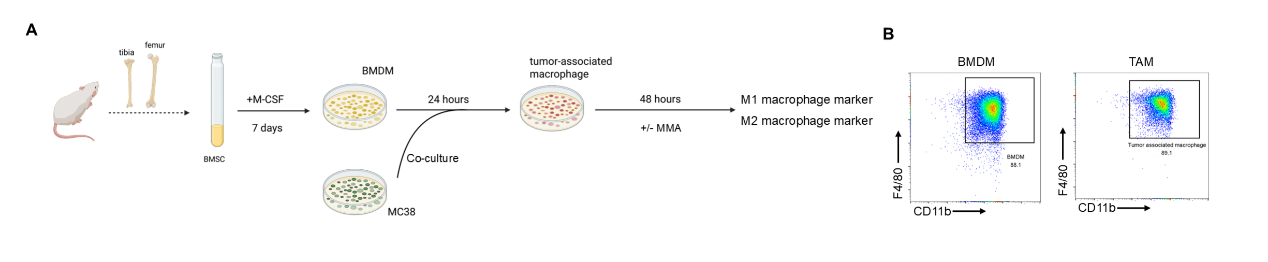


**Figure S4. Construction and verification of the *in vitro* tumor-associated macrophage (TAM).** **(A)** Schematic illustration of the experimental workflow. Bone marrow cells were isolated from the tibia and femur of mice and differentiated into bone marrow-derived macrophages (BMDMs) in the presence of M-CSF for 7 days. BMDMs were then co-cultured with MC38 colon cancer cells for 24 hours to generate TAM-like cells, followed by treatment with or without MMA for 48 hours prior to marker analysis. **(B)** Flow cytometric analysis characterizing the purity of BMDMs and TAMs. Cells were stained with macrophage surface markers CD11b and F4/80. n=3 for each group.
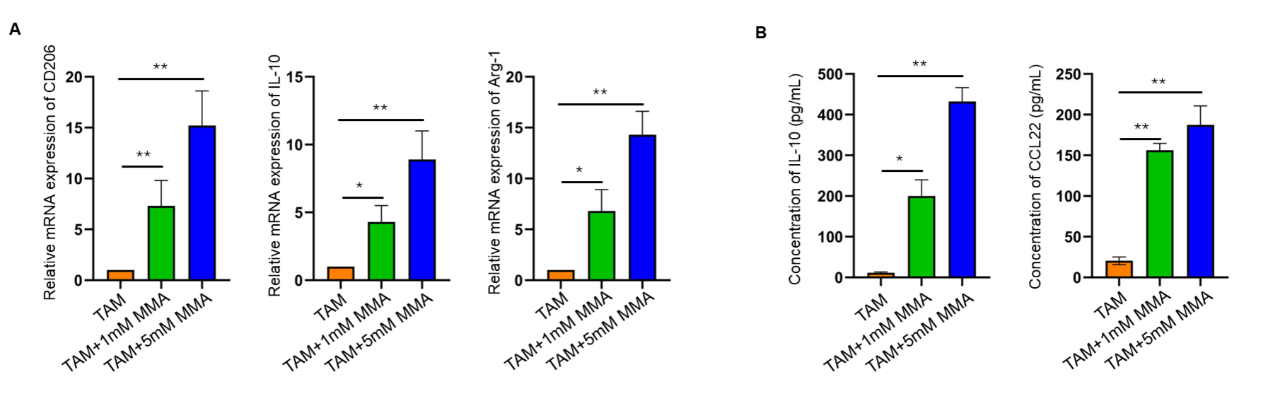
**Figure S5. MMA dose-dependently promotes M2 macrophage polarization *in vitro*.** **(A)** Relative mRNA expression levels of typical M2 macrophage markers (CD206, IL-10, and Arg-1) in TAMs treated with 0, 1 mM, or 5 mM MMA. **(B)** Concentrations of secreted cytokines (IL-10 and CCL22) in the cell culture supernatants, as measured by ELISA. Data are presented as mean ± SEM. **P* < 0.05, ***P* < 0.01.
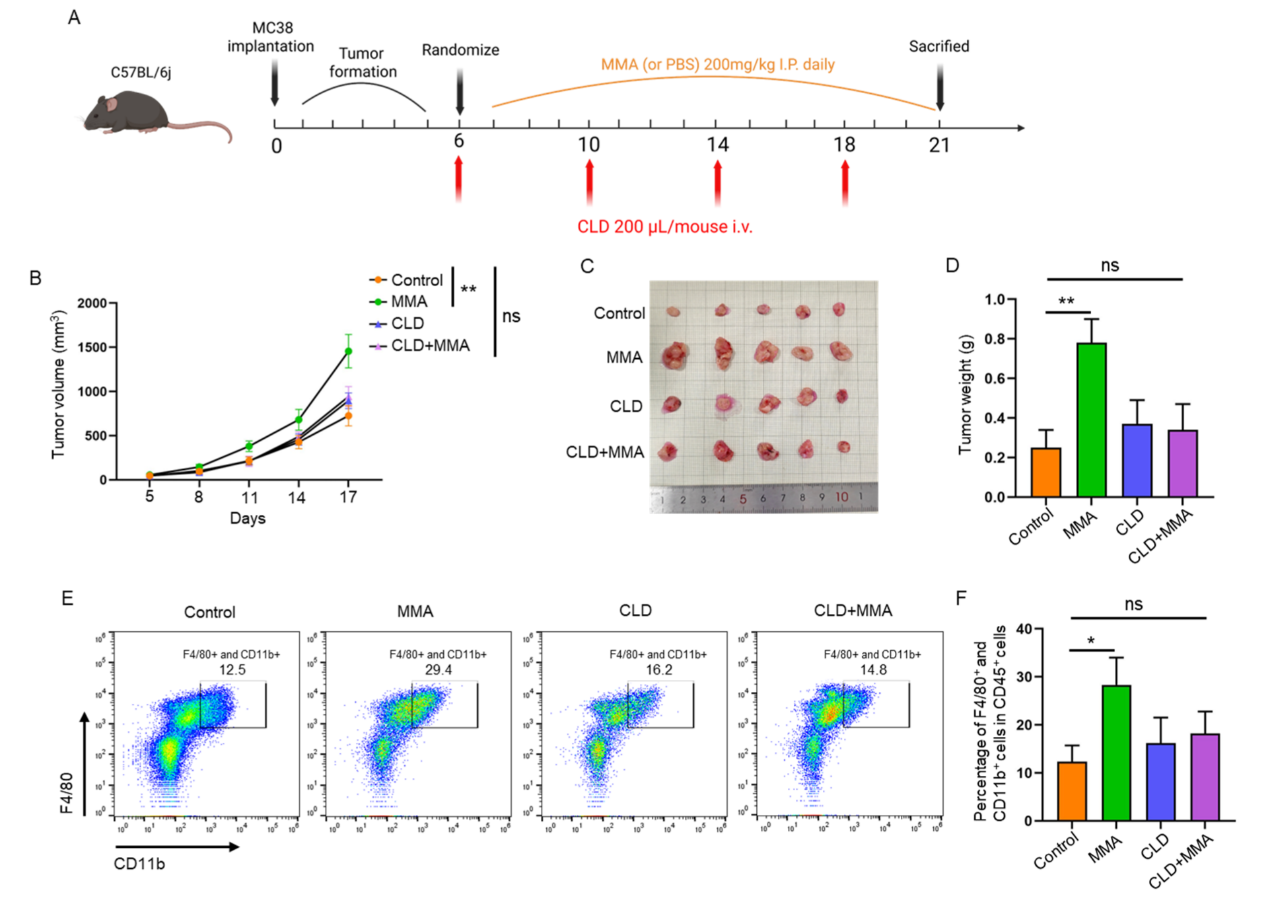


**Figure S6. Systemic macrophage depletion abrogates the MMA-induced promotion of tumor growth *in vivo*.** **(A)** Schematic illustration of the experimental design. C57BL/6J mice bearing MC38 subcutaneous tumors were randomized and treated with MMA (200 mg/kg, i.p., daily) or vehicle control, with or without systemic macrophage depletion using intravenous clodronate liposomes (CLD, 200 μL/mouse) at the indicated time points. **(B)** Tumor growth curves depicting the tumor volumes of the indicated groups measured over time. **(C)** Representative macroscopic images of the excised MC38 tumors from each group at the experimental endpoint. **(D)** Quantitative analysis of the final tumor weights at the endpoint. **(E)** Representative flow cytometry plots showing the infiltration of tumor-associated macrophages (TAMs) in the tumor microenvironment. **(F)** Statistical quantification of the percentage of TAMs (defined as F4/80^+^ and CD11b^+^ cells) within the total live CD45^+^ leukocyte population. Data are presented as mean ± SEM (*n* = 5 mice per group). Statistical significance was determined by one-way ANOVA with Tukey's post hoc test. **P* < 0.05, ***P* < 0.01; ns, not significant.


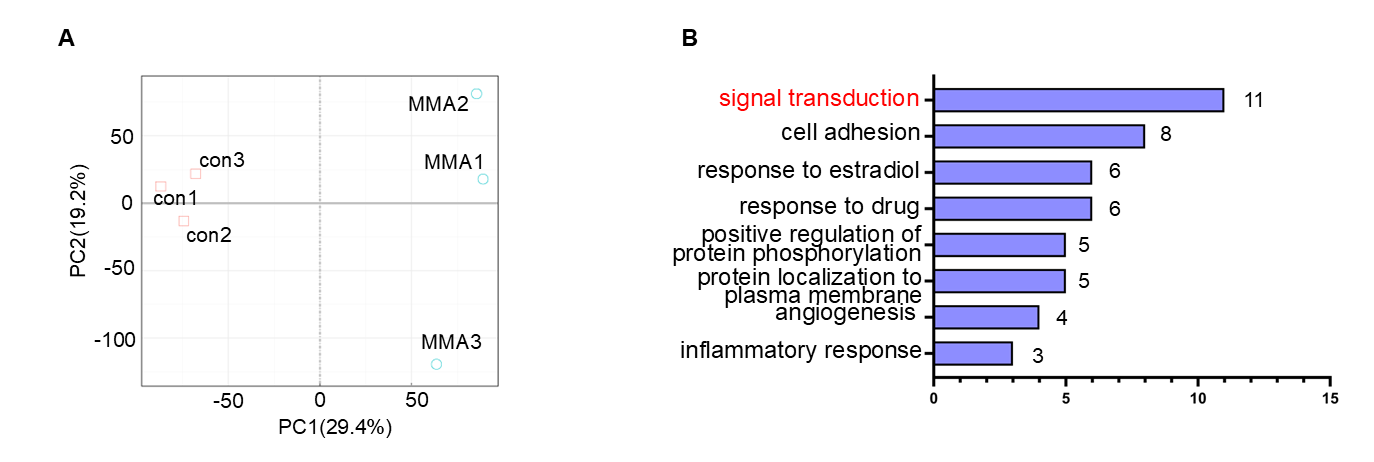


**Figure S7. RNA-seq analysis reveals altered signaling pathways in MMA-treated TAMs.** **(A)** Principal component analysis (PCA) plot of RNA-sequencing data showing the distinct separation between the control group (con) and the MMA-treated group (MMA) (n=3 for each group). **(B)** Gene Ontology (GO) enrichment analysis of the differentially expressed genes (DEGs). The bar chart displays the top enriched biological processes. The numbers adjacent to the bars indicate the count of enriched genes in each pathway. The "signal transduction" pathway (highlighted in red) was identified as a significantly regulated process.


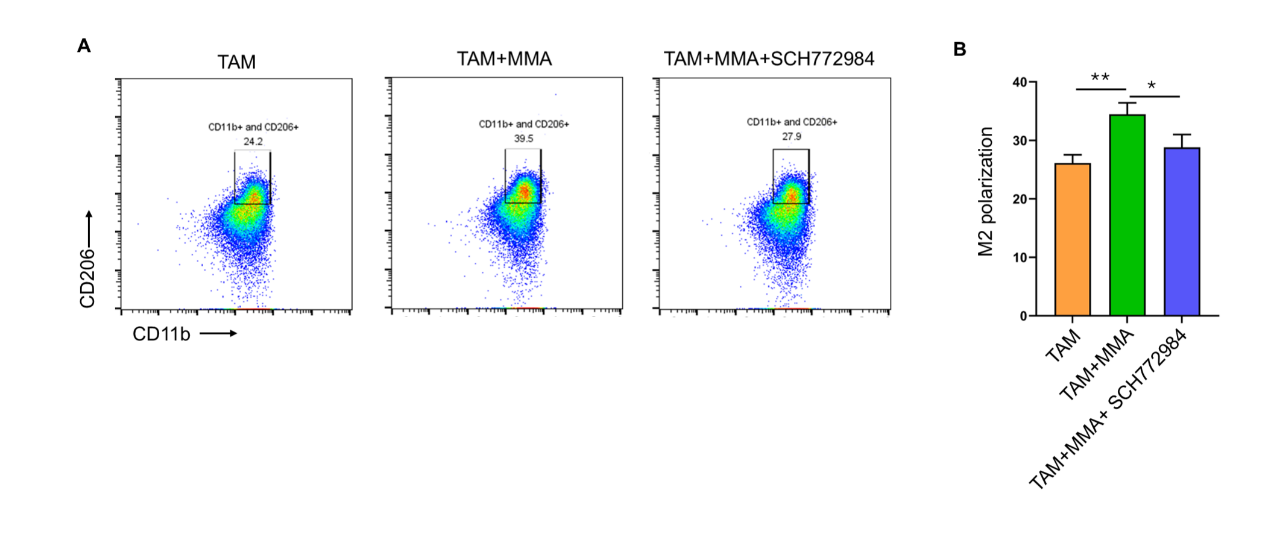
**Figure S8. SCH772984 reverses MMA-induced M2 polarization of TAMs.** (A) Representative flow cytometry plots showing the expression of CD11b and CD206 in TAMs. Cells were treated with MMA alone or in combination with the inhibitor SCH772984. (B) Quantification of M2 polarization (percentage of CD11b and CD206 positive cells) across the indicated groups. n=3 for each group. Data are presented as mean with SEM. Statistical significance was analyzed using one-way ANOVA. *, *P* < 0.05; **, *P* < 0.01.


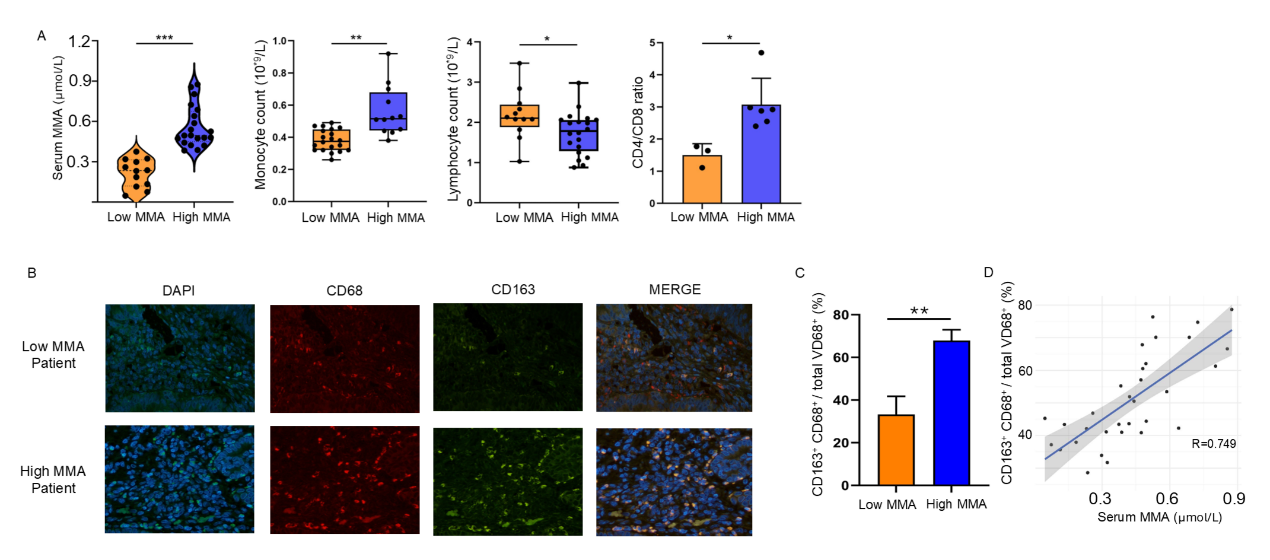


**Figure S9. MMA is closely associated with poor clinical prognosis and tumor-associated macrophage infiltration in CRC patients.** **(A)** Analysis of clinical blood samples showing that patients with High serum MMA levels exhibit significantly higher monocyte counts and altered CD4/CD8 ratios compared to the Low MMA group. **(B)** Representative immunofluorescence images of human CRC tissue sections stained for CD68 (red, macrophage marker) and CD163 (green, M2 marker). **(C)** Quantitative analysis showing a significantly higher percentage of M2 macrophages in the High MMA group. **(D)** Linear regression analysis revealing a strong positive correlation (R = 0.749) between serum MMA concentration and the density of infiltrating M2 macrophages in tumor tissues. *, *P* < 0.05; **, *P* < 0.01, ***, *P* < 0.001.


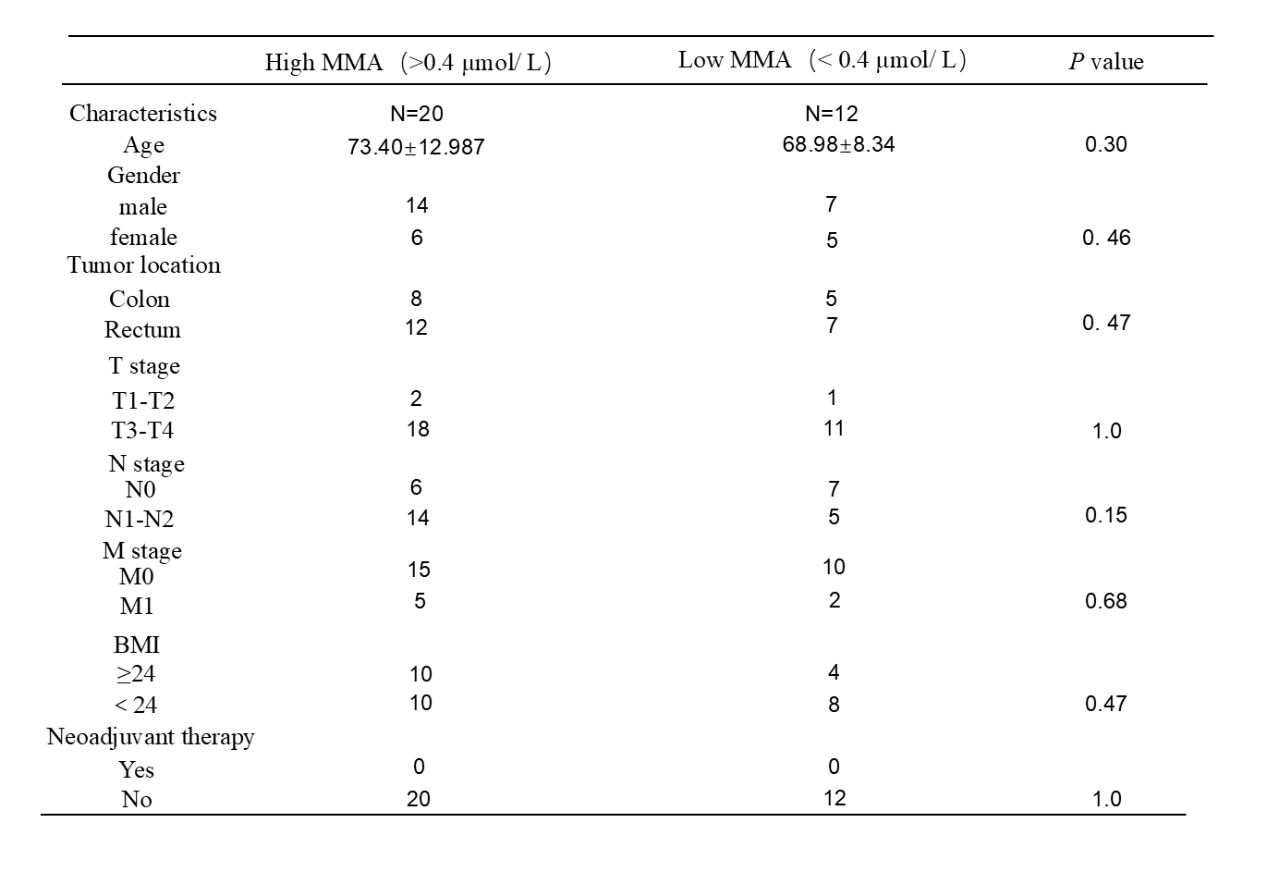


**Table S1. Comparison of clinical characteristics according to serum MMA Levels in CRC patients.**
